# A case report of prostate cancer presenting as a symptomatic pelvic mass mimicking lymphoma

**DOI:** 10.1016/j.ijscr.2020.11.042

**Published:** 2020-11-11

**Authors:** Zied Mahjoubi, Mokhtar Bibi, Houssem Hedhli, Yassine Ouanes, Sami Ben Rhouma, Nouira Yassine

**Affiliations:** Department of Urology, Hospital La Rabta, 1007, Tunis, Tunisia

**Keywords:** Metastatic prostate cancer, Pelvic mass, Lymphoma, Orchidectomy, Androgen deprivation, Case report

## Abstract

**Introduction:**

Metastatic prostate cancer is a relatively common condition in elderly men. The aim of this paper is to report a rare case of metastatic prostate cancer presenting as a large pelvic mass mimicking lymphoma and to discuss its management.

**Presentation of Case:**

An 85-year-old patient was complaining of lower urinary tract symptoms, and significant pelvic swelling. On physical examination, a 15-cm pelvic mass and lumpy prostate were found. Laboratory investigations revealed elevated PSA levels (PSA=300g/mL). CT scan showed a large lobulated necrotic pelvic mass and multiple swollen lymphnodes. The diagnosis of an atypical pelvic lymphoma associated with a metastatic prostate cancer was first evoked. An ultrasonography-guided biopsy of the mass was carried. The immunohistochemical study confirmed prostate adenocarcinoma. A bilateral orchiectomy was performed. A follow up of 3 months showed a decrease of PSA without shrinkage of the mass.

**Discussion:**

A metastatic prostate cancer presenting as a compressive pelvic mass mimicking other diagnosis such as lymphomas, is a rare circumstance of discovery. In our case, atypical pelvic lymphoma was evoked based on the association of multiple retroperitoneal enlarged lymphnodes. Histological findings of the biopsy rectified the diagnosis and confirmed prostate adenocarcinoma. The main treatment for such metastatic prostate cancers remains hormone therapy. Some authors reported external beam radiotherapy in order to reduce prostate volume.

**Conclusion:**

Prostate cancer should be considered in the assessment of large pelvic masses. Digital rectal examination and testing PSA levels can lead to the diagnosis. After histological confirmation, the androgen deprivation is the main treatment.

## Highlights

• Metastatic prostate cancer presenting as a large pelvic mass mimicking lymphoma.
• CT scan confirmed a large lobulated necrotic pelvic and extraperitoneal mass.
• Bilateral orchidectomy was performed.
• The main treatment for metastatic prostate cancer is hormone therapy.
• External beam radiotherapy is proven to help decreasing tumor volume.

## 1 Introduction

Prostate cancer is the second most common male cancer and remains a leading cause of cancer death worldwide. It is a heterogeneous disease that displays a various range of clinical behavior, from a slow-growing tumor of no clinical significance to an aggressive, metastatic and lethal disease. The aim of this paper is to report a rare case of metastatic prostate cancer presenting as a large symptomatic pelvic mass mimicking lymphoma and to discuss its management. This work has been reported in line with the SCARE 2018 criteria [1].

## 2 Presentation of case

An 85-year-old patient was referred to our emergency department complaining of asthenia, loss of appetite, lower urinary tract symptoms, and significant pelvic painful swelling. He had no history of chronic disease or surgery.

On physical examination, the abdomen was distended, owing to an enlarged pelvic swelling with ill-defined margins, fixed to the abdominal wall and measuring 15 cm. Digital rectal exam showed an enlarged and lumpy prostate. Laboratory investigations revealed hypochromic and microcytic anemia (Hb = 9 g/dcl) and elevated serum PSA levels (PSA = 300 g/mL).

Ultrasonography revealed a large heterogeneous lobulated ill-defined mass located in the left lower abdominal quadrant. It measured 15 cm and appeared to be partially liquefied by necrosis. The liver, spleen, and kidneys showed no abnormalities.

**Fig. 1:**
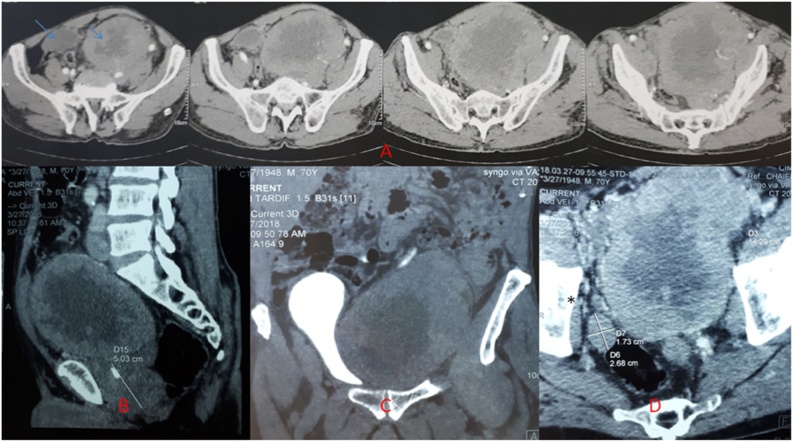
CT scan images showing a large lobulated necrotic pelvic and extraperitoneal mass with multiple swollen lomboaortic and iliac lymph nodes.

Computerized tomography (CT) scan confirmed this large lobulated necrotic pelvic and extraperitoneal mass with medial displacement of the bladder, causing mild bilateral hydroureteronephrosis. The mass was adjacent to a calcified and enlarged prostate. Multiple swollen lomboaortic and iliac lymph nodes, measuring from 26 to 39 mm, were also noted (Fig. 1A–D). Bone scan showed suspicious lytic lesions of the right ischium and the posterior arch of the two upper sacral vertebrae.

**Fig. 2:**
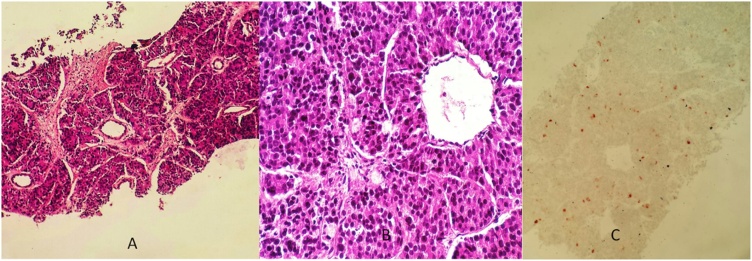
A and B: Pathological report demonstrating eosinophilic cells with large hyperchromatic nuclei containing visible nucleoli. C: Immunohistochemical study showing eosinophilic cells containing focal cytoplasmic with PSA staining, confirming a prostate adenocarcinoma Gleason 9.

The case was presented at a multi-disciplinary team meeting including the urologist, general surgeon, pathologist and radiologist. Due to multiple retroperitoneal enlarged lymphnodes, we have first evoked the diagnosis of an atypical pelvic lymphoma associated with a metastatic prostate cancer. An ultrasonography-guided biopsy was carried. After the immunohistochemical study, the pathological report demonstrated eosinophilic cells with large hyperchromatic nuclei containing visible nucleoli (Fig. 2A, B) and focal cytoplasmic with PSA staining, confirming a prostate adenocarcinoma Gleason 9 (Fig. 2C).

A bilateral orchidectomy was hence performed. The postoperative course was uneventful. A follow up of 3 months showed an improvement in performance status and a decrease of PSA down to 60 ng/mL without shrinkage of the pelvic mass on physical examination.

## 3 Discussion

Metastatic prostate cancer is a relatively common disease of elderly men. It is routinely suspected by feeling a lumpy prostate on digital rectal exam and finding elevated PSA levels in blood tests. The diagnosis is confirmed by histological examination of tissue samples of the prostate biopsy. Metastatic lesions are mainly assessed by performing pelvic MRI and bone scan. However, a metastatic prostate cancer revealed by a compressive symptomatic pelvic mass mimicking other diagnosis such as lymphomas, is an unusual circumstance of discovery.

Indeed, urologists may be confronted in their daily practice to patients suffering from large pelvic symptomatic masses. The differential diagnoses include a variety of primary or secondary pelvic tumors (bladder carcinoma, seminoma, germinal tumors, neurogenic tumors, benign teratoma, liposarcoma, smooth muscle tumors, fibrous malignant histiocytoma, hemangiopericytoma), inflammatory processes (actinomycosis, pelvic tuberculosis), idiopathic retroperitoneal fibrosis, and lymphoproliferative disorders. In our case, a symptomatic pelvic mass turned out to be a Gleason 9 prostate adenocarcinoma. To our knowledge, less than 30 cases were reported in the literature. The first case of a giant prostate cancer appearing as abdominal mass, was presented by Chait et al. in 1966 [2]. In Japan, a total of 21 cases were reported. The mean age of patients was 70.8 years. Most Patients were complaining of abdominal pain and lower urinary tract symptoms. PSA varied from 170 mg/mL to 27,000 mg/mL. The mean follow up was 10.2 months. More than 80 % of cases were moderately and well-differentiated adenocarcinoma [3]. Another study showed a giant neuroendocrine tumor of the prostate with pulmonary and hepatic metastasis at diagnosis [4].

Imaging plays a pivotal role both in the diagnostic phase of these pelvic masses and in the recognition of their potential complications such as compressing of the adjacent structures. MRI is indicated to determine the involvement of the seminal vesicles, bladder, and pelvic side walls, as well as pelvic and abdominal lymphnodes, for staging purposes [4]. In our case, CT scan findings were sufficient to characterize the tumor. Atypical pelvic lymphoma was evoked based on the association of multiple retroperitoneal lymphnodes [5]. Histological findings of the biopsy rectified the diagnosis and confirmed a metastatic prostate cancer that has already spread to bone and to multiple nearby and distant lymphnodes.

The main treatment for metastatic prostate cancer is hormone therapy. Ze Ondo C, et al. reported a giant prostate cancer treated by LHRH agonists with a significant decrease of the tumor volume [4]. Some authors reported external beam radiotherapy in order to reduce prostate volume. Kanda reported an excellent local control with 96.7 % shrinkage of tumor after external beam radiation therapy (60 Gy) to the prostate [6]. Zechmann et al. has compared the changes of prostate gland volume with and without androgen deprivation after intensity modulated radiotherapy on 39 patients. The authors found that patients undergoing intensity modulated radiotherapy, showed definite prostate shrinkage and that there was no adding effect of androgen deprivation on prostate volume [7]. In our case, a surgical castration by bilateral orchiectomy, was performed. The outcome was a remarkable improvement of the performance status a decrease of PSA down to 60 ng/mL but without decrease of the pelvic mass on physical examination.

## 4 Conclusion

Prostate cancer should be considered in the assessment of large pelvic masses. Digital rectal examination and testing PSA levels can lead to the diagnosis. After histological confirmation, the androgen deprivation is the main treatment and radiotherapy is proven to help decreasing tumor volume.

## Declaration of competing interest

None to be declared.

## Funding

No source to be stated.

## Ethical approval

Ethical approval is not necessary for case report in our locality

## Consent

Written informed consent was obtained from the patient for publication of this case report and accompanying images. A copy of the written consent is available for review by the Editor-in-Chief of this journal on request.

## Author contribution

Mahjoubi Zied: Writing the manuscript, literature review, final approval of the manuscript and follow up.

Mokhtar Bibi: Surgeon performing the orchidectomy.

Mokhtar Bibi, Houssem Hedhli, Yassine Ouanes, Sami Ben Rhouma, Nouira Yassine: final approval of the manuscript.

Corresponding Author: Mahjoubi Zied

## Registration of research studies

Not applicable

## Guarantor

Mahjoubi Zied is the Guarantor.

## Provenance and peer review

Not commissioned, externally peer-reviewed.
